# Feasibility of ultra-low flow rate coronary CT angiography using photon-counting detector CT: a prospective randomized trial

**DOI:** 10.1186/s41747-026-00677-3

**Published:** 2026-02-24

**Authors:** Shuangxiang Lin, Cuiliu Liu, Yalan Zhou, Qinlan Chen, Shuyue Wang, Jiaxing Wu, Xinhong Wang, Jianzhong Sun

**Affiliations:** 1 Department of Radiology, The Second Affiliated Hospital, Zhejiang University School of Medicine, Hangzhou, China; 2 Siemens Healthineers, Siemens, Shanghai, China

**Keywords:** Contrast media, Coronary CT angiography, Image quality, Photon-counting detector CT, Ultra-low contrast flow rate

## Abstract

**Objective:**

This study evaluates the feasibility of photon-counting detector CT (PCD-CT)-based coronary CT angiography (CCTA) using ultra-low flow contrast rate while maintaining diagnostic image quality.

**Materials and methods:**

In this prospective trial, 292 patients underwent CCTA assigned to one of three protocols: ultra-low (1.5–1.8 mL/s) or routine (4.0–5.0 mL/s) contrast injection with PCD-CT, or routine injection with EID-CT. All scans utilized a high-pitch prospective electrocardiogram-triggering acquisition. PCD-CT images were reconstructed at 45 keV (ultra-low) or 60 keV (routine). Objective image quality was quantitatively assessed by measuring vessel attenuation, signal-to-noise ratio (SNR), and contrast-to-noise ratio (CNR). Subjective image quality parameters (vascular contrast, image noise, artifacts, and vessel clarity) were independently evaluated by two blinded readers using a 4-point Likert scale (1: non-diagnostic; 2: adequate; 3: good; 4: excellent).

**Results:**

Objective image quality demonstrated comparable attenuation, CNR, and SNR in proximal coronary segments across all groups (all *p* > 0.05). The ultra-low PCD-CT protocol significantly lowers attenuation in the distal LAD (373.20 ± 49.58 HU) compared to routine protocols (PCD-CT: 393.52 ± 49.38 HU; EID-CT: 396.72 ± 47.55 HU; *p* = 0.01). While distal vessel clarity scores were modestly reduced in distal vessel clarity (ultra-low PCD-CT: 2.91 ± 0.81 *versus* routine PCD-CT: 3.58 ± 0.50 *versus* routine EID-CT: 3.54 ± 0.50; *p* < 0.01).

**Conclusion:**

For patients with difficulty establishing venous access routes, ultra-low contrast agent flow rates in PCD-CT maintain objective image quality comparable to that of standard protocols, with acceptable diagnostic performance despite slight reductions.

**Relevance statement:**

Photon-counting detector CT (PCD-CT) maintains objective coronary CT angiography image quality comparable to standard protocols even at ultra-low contrast flow rates (1.5–1.8 mL/s), offering a clinically acceptable and safer alternative for patients with challenging venous access.

**Key Points:**

- First validation of ultra-low flow contrast rate CCTA using photon-counting CT (PCD-CT).
- Ultra-low flow rates maintain objective image quality (CNR/SNR) *versus* routine protocols.
- PCD-CT enables 50% contrast reduction without diagnostic compromise.

**Graphical Abstract:**

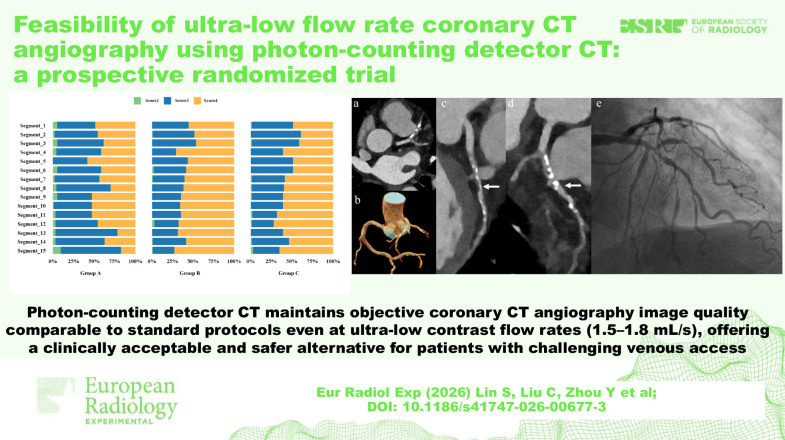

## Background

Coronary CT angiography (CCTA) is an established, non-invasive modality for evaluating coronary artery disease [1, 2]. High-quality CCTA necessitates sufficient intravascular contrast enhancement, typically achieved through high-flow rates and volumes of iodinated contrast agents [3, 4]. However, administering large contrast volumes at rapid injection rates poses significant challenges for patients with difficult venous access, such as those with fragile veins or limited vascular sites [5, 6]. These patients face increased risks of contrast extravasation, vein rupture, and associated complications, which can compromise patient safety and lead to suboptimal imaging that may require repeat scans and additional radiation exposure [7, 8].

Advancements in computed tomography (CT) technology, particularly photon-counting detector CT (PCD-CT), offer promising solutions to these challenges [9, 10]. PCD-CT employs detectors that directly convert X-ray photons into electrical signals, delivering superior spatial resolution, reduced electronic noise, and enhanced dose efficiency compared to conventional energy-integrating detector CT (EID-CT) systems [11, 12]. By using energy-specific thresholds, each photon is assigned to a specific energy bin, enabling the acquisition of multi-energy information [13, 14]. Emerging clinical evidence has demonstrated the potential of PCD-CT to reduce contrast volume requirements in coronary CT angiography (CCTA) without compromising diagnostic image quality [15–17]. However, while most current CCTA protocols employ flow rates exceeding 3 mL/s, there remains limited research on optimizing protocols for ultra-low flow conditions (< 2 mL/s). De Santis et al provided important evidence that even conventional EID-CT can achieve diagnostic coronary enhancement at low iodine delivery rates (0.4–1.0 gI/s) using low-energy virtual monochromatic imaging (VMI) [18]. Nevertheless, to date, no study has specifically investigated the performance of PCD-CT under ultra-low flow rate conditions. This knowledge gap is particularly relevant for patients with fragile or compromised venous access, for whom high-flow contrast injections pose safety risks. The implementation of optimized ultra-low flow protocols using PCD-CT could therefore minimize the risk of contrast extravasation, reduce patient discomfort, and enhance safety in these vulnerable populations.

In this study, we aim to evaluate the feasibility and efficacy of using ultra-low contrast agent injection rates and dosages in CCTA performed with PCD-CT for patients. We hypothesize that PCD-CT can maintain high image quality and diagnostic accuracy even at ultra-low iodinated contrast injection rates due to its enhanced detector sensitivity and improved SNR.

## Materials and methods

### Study design and patients

This prospective, randomized controlled trial evaluated the feasibility and effectiveness of reducing contrast agent flow rates in CCTA using both EID-CT and PCD-CT systems. The study was approved by the Institutional Review Board (IR2024488) and conducted in accordance with the Declaration of Helsinki. All participants provided written informed consent.

**Fig. 1:**
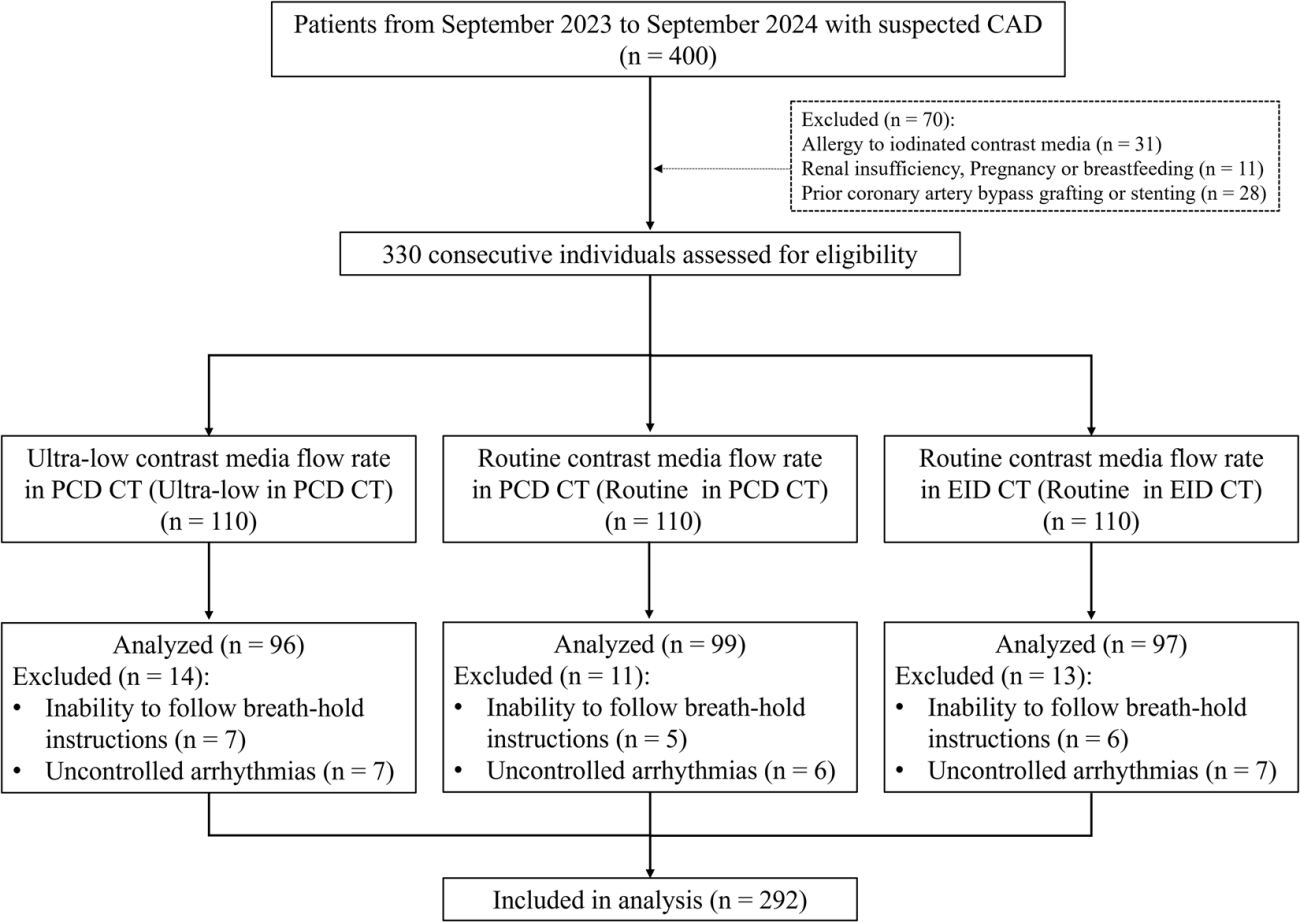
Flowchart of study population. PCD-CT, Counting detector CT; EID-CT, Energy-integrating detector CT

Between September 2023 and September 2024, consecutive patients referred for clinically indicated CCTA at our institution were screened for eligibility. Inclusion criteria were adults aged 18 years or older with suspected or known CAD requiring diagnostic evaluation via CCTA. Exclusion criteria included known allergy to iodinated contrast media, renal insufficiency, pregnancy or breastfeeding, inability to follow breath-hold instructions, uncontrolled arrhythmias or atrial fibrillation, and coronary interventions. The study flow chart is shown in Fig. 1.

### Contrast administration

Eligible patients were randomly assigned in a 1:1:1 ratio to one of three groups evaluating contrast injection protocols for CCTA on PCD-CT or EID-CT systems. The randomization was performed using computer-generated sequences with permuted blocks of three, and allocation concealment was ensured with sealed envelopes that were opened at the time of scanning. Three contrast injection protocols were designed to compare the effects of flow rate and detector technology: ultra-low flow PCD-CT, standard flow PCD-CT, and standard flow EID-CT. Ultra-low flow rate in PCD-CT received 15–25 mL (0.3 mL/kg) of contrast media, injected at a flow rate of 1.5–1.8 mL/s, followed by a 40 mL saline flush at the same rate. Standard Flow Rate in EID-CT and Standard Flow Rate in PCD-CT each received 40–50 mL (0.8 mL/kg) of contrast media injected at a flow rate of 4.0–5.0 mL/s, followed by a 40 mL saline flush at the same rate. All groups were administered an iodine-based contrast agent (Omnipaque 350, iodine concentration 350 mg/mL; GE Healthcare).

### CCTA data acquisition and image reconstruction

All coronary CT angiography examinations were performed using dual-source CT systems: an energy-integrating detector (EID) system (SOMATOM Force) or a spectral photon-counting detector (PCD) system (NAEOTOM Alpha; both Siemens Healthineers). All patients underwent ECG-gated high-pitch acquisition (pitch 3.2) in the supine position. Phase selection was automated based on heart rate: systolic phase (T-wave synchronization) for > 75 bpm and diastolic phase for ≤ 75 bpm. The standard PCD-CT group was reconstructed using the manufacturer-recommended default virtual monoenergetic imaging level of 60 keV. Ultra-low in PCD-CT images were reconstructed using a virtual monoenergetic algorithm at 45 keV (Bv36 kernel, 0.6-mm slice thickness/0.4-mm increment) based on prior optimization studies [19, 20]. All patients received 400 mg of sublingual glyceryl trinitrate as part of pre-procedural preparation, without the administration of β-blockers. Technical specifications and acquisition parameters are comprehensively detailed in Table 1.

**Table 1:** The detailed image acquisition and CT system specifications

	Ultra-low in PCD-CT	Routine in PCD-CT	Routine in EID-CT
Scan Mode	Prospectively ECG-triggered FlashSpiral mode	Prospectively ECG-triggered FlashSpiral mode	Prospectively ECG-triggered FlashSpiral mode
Voltage (kVp)	140	140	70–90
Effective tube current (mAs)	-	-	370–450
IQ level	100	80	-
Collimation (mm)	144 × 0.4	144 × 0.4	192 × 0.6
Pitch	3.2	3.2	3.2
Reconstruction algorithm	QIR 3	QIR 3	ADMIRE 3
Kernel	Bv40	Bv40	Bv40
Slice thickness (mm)	0.6	0.6	0.6
Increasements (mm)	0.4	0.4	0.4
Matrix size (mm × mm)	Auto-Matrix	Auto-Matrix	512 * 512
Contrast agent volume (mL)	15–20	45–50	45–50
Contrast agent flow rate (mL/s)	1.5–1.8	4.5–5	4.5–5

*IQ level* Image quality level; *QIR* Quantum iterative reconstruction, *ADMIRE* Advanced modeled iterative reconstruction

### Estimation of radiation dose

The effective dose of CCTA was calculated by multiplying the dose-length product by a conversion coefficient for the chest (*k* = 0.014 mSv·mGy⁻¹·cm⁻¹), in accordance with the European Working Group for Guidelines on Quality Criteria in CT [21]. Additionally, a cardiac-specific *k*-factor (*k* = 0.026 mSv·mGy⁻¹·cm⁻¹) was employed for specialized dose management in cardiac imaging [22].

### Evaluation of objective image quality

A radiologist with 6 years of experience in cardiovascular diagnosis performed measurements on axial images. Mean CT attenuation was measured in the ascending aorta, left main artery (LM), proximal and distal segments of the left anterior descending artery (LAD), left circumflex artery (LCX), and right coronary artery (RCA), as well as in adjacent adipose tissue. Circular regions of interest (ROIs) were carefully placed within the vessel lumen, avoiding areas that were too small or too large to prevent partial volume effects and to exclude vessel edges, calcification, plaque, and artifacts. ROI sizes in the ascending aorta and proximal and distal coronary segments were approximately 300, 5, and 3 mm², respectively. Image noise was defined as the standard deviation of attenuation within an ROI placed in the ascending aorta. Contrast-to-noise ratio (CNR) and signal-to-noise ratio (SNR) were calculated using standard formulas:

$${{\rm{CNR}}}=\frac{{{{Mean\; Attenuation}}}_{{{coronary\; artery}}}-{{{Mean\; Attenuation}}}_{{{adipose\; tissue}}}}{{{\rm{Image\; Noise}}}}$$

$${{\rm{SNR}}}=\frac{{{Mean\; Attenuation}}}{{{\rm{Image\; Noise}}}}$$

### Evaluation of subjective image quality

**Fig. 2:**
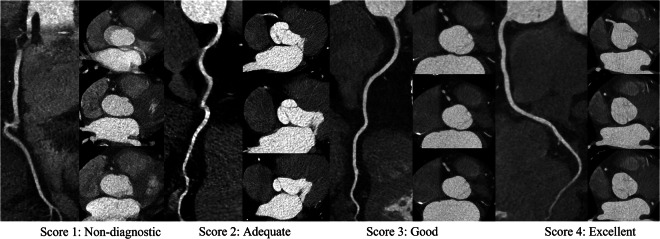
The axial plane and representative curved multiplanar reformation images illustrate examples of the 4-point Likert scale, which assesses the degree of motion artifacts and vascular visualization quality in the right coronary arteries. Score 1 indicates non-interpretable image quality characterized by severe artifacts and poor distal vessel filling, hindering accurate stenosis quantification; Score 2 reflects adequate image quality with moderate artifacts and acceptable moderate distal vessel filling for diagnostic purposes; Score 3 denotes good image quality with minor artifacts and well-filled distal vessels; Score 4 represents excellent image quality devoid of motion artifacts, exhibiting full distal vessel filling

Two experienced cardiovascular radiologists (each with > 10 years of experience), blinded to group assignments, independently evaluated all studies in two separate reading sessions. Image quality was assessed based on coronary artery contrast, image noise, beam-hardening artifacts, distal vessel clarity of the LAD, LCX, and RCA, and overall image quality using 4-point Likert scale (1: non-diagnostic; 2: adequate; 3: good; 4: excellent)(Fig. 2). Discrepancies were resolved in a third consensus review held at least three weeks after the initial sessions to minimize recall bias. The overall image quality score was used to evaluate the impact of the reduced contrast dose on the assessability of each coronary segment according to the American Heart Association 15-segment model [23].

### Statistical analysis

Statistical analyses were performed using R version 4.0.5 (R Foundation for Statistical Computing). Continuous variables are presented as mean ± standard deviation or median with interquartile range, as appropriate. Categorical variables are summarized as counts and percentages. The normality of continuous variables was assessed using the Shapiro-Wilk test. For comparisons across the three groups, one-way analysis of variance (ANOVA) or the Kruskal-Wallis test was employed for continuous variables with normal or non-normal distributions, respectively. If a statistically significant difference was found, post hoc analyses (Tukey’s test for ANOVA or Dunn’s test with Bonferroni correction for Kruskal-Wallis) were conducted for pairwise comparisons. The chi-square (*χ*²) test or Fisher’s exact test was used to compare categorical variables, as appropriate. Interobserver agreement was assessed using intraclass correlation coefficients (ICCs). Based on the 95% confidence interval (CI) of the ICC estimate, the reliability was determined to be poor (< 0.5), moderate (0.5–0.75), good (0.75–0.9), and excellent (> 0.90). A *p*-value less than 0.05 was considered statistically significant.

## Results

### The patient characteristics

A total of 292 patients were enrolled and divided into three groups: ultra-low rate in PCD-CT (*n* = 96), routine rate in PCD-CT (*n *= 97), and routine rate in EID-CT (*n* = 99). Demographic and clinical characteristics are summarized in Table 2. There were no significant differences among the groups in age, body mass index, or sex distribution (*p *> 0.05 for all). Cardiovascular risk factors and CAD-RADS scores were similarly distributed across groups. The mean Agatston calcium scores were comparable among the groups (*p* = 0.21).

**Table 2:** Patient characteristics in different groups

Variables	Total	Ultra-low in PCD-CT	Routine in PCD-CT	Routine in EID-CT	*p*-value
	(*n* = 292)	(*n* = 96)	(*n* = 99)	(*n* = 97)	
Demographics					
Age (years)	54.63 ± 14.24	53.64 ± 14.54	55.75 ± 14.78	54.48 ± 13.42	0.58
Body mass index (kg/m^2^)	21.23 ± 3.53	21.55 ± 3.29	21.08 ± 3.49	21.06 ± 3.81	0.55
Sex (Female, *n*, %)	138 (47.26)	47 (48.96)	47 (47.47)	44 (45.36)	0.88
Cardiovascular risk factors					
Hypertension (*n*, %)	154 (52.74)	45 (46.88)	57 (57.58)	52 (53.61)	0.32
Diabetes (*n*, %)	146 (50.00)	49 (51.04)	46 (46.46)	51 (52.58)	0.67
Hypercholesterolemia (*n*, %)	145 (49.66)	48 (50.00)	46 (46.46)	51 (52.58)	0.69
Current smoke (*n*, %)	140 (47.95)	46 (47.92)	45 (45.45)	49 (50.52)	0.78
CCTA data					
Calcium Scoring by Agatston	183.03 ± 161.64	159.47 ± 166.43	190.87 ± 159.47	198.35 ± 158.00	0.21
CAD-RADS					0.54
0	63 (21.58)	24 (25.00)	22 (22.68)	17 (17.17)	
1	68 (23.29)	26 (27.08)	19 (19.59)	23 (23.23)	
2	66 (22.60)	19 (19.79)	27 (27.84)	20 (20.20)	
3	63 (21.58)	17 (17.71)	19 (19.59)	27 (27.27)	
4	32 (10.96)	10 (10.42)	10 (10.31)	12 (12.12)	
DLP (mGy·cm)	56.05 ± 28.18	69.64 ± 16.88	60.29 ± 18.01	40.57 ± 15.95	< 0.01
Effective dose (mSv)	0.78 ± 0.39	0.97 ± 0.23	0.84 ± 0.25	0.56 ± 0.22	< 0.01
Cardiac-specific effective dose (mSv)	1.46 ± 0.73	1.81 ± 0.44	1.57 ± 0.47	1.05 ± 0.41	< 0.01
Mean HR during scan (bpm)	78.26 ± 8.43	76.14 ± 8.07	79.56 ± 8.93	79.11 ± 8.28	0.42

*CAD-RADS* Coronary Artery Disease Reporting and Data System, *DLP* Dose-length product, Effective Dose = DLP*0.014, Cardiac-specific effective dose = DLP*0.026

The effective dose differed significantly among the groups (*p* < 0.01), with the ultra-low dose PCD-CT group demonstrating the highest radiation exposure among the cohorts, with a dose-length product (DLP) of 69.64 ± 16.88 mGy·cm, an effective dose of 0.97 ± 0.23 mSv, and a cardiac-specific effective dose of 1.81 ± 0.44 mSv.

### Objective image quality measurements

Table 3 shows the results of objective image quality assessment. For attenuation measurements, The distal LAD attenuation was significantly lower in the ultra-low PCD-CT group (373.20 ± 49.58 HU) than in the routine PCD-CT (393.52 ± 49.38 HU) and routine EID-CT (396.72 ± 47.55 HU) groups (*p *= 0.01). Similarly, the distal LCX attenuation was significantly reduced in the ultra-low PCD-CT group (374.33 ± 46.83 HU) compared with the routine PCD-CT (400.70 ± 50.04 HU) and routine EID-CT (389.84 ± 49.07 HU) groups (*p* < 0.01). For CNR, the distal LAD CNR was significantly lower in the ultra-low PCD-CT group (25.73 ± 5.43) than in the routine PCD-CT (27.55 ± 5.11) and routine EID-CT (26.24 ± 5.33) groups (*p* = 0.05). In contrast, the proximal RCA CNR was significantly higher in the ultra-low PCD-CT group (30.65 ± 5.28) compared with the routine PCD-CT (28.96 ± 5.23) and routine EID-CT (29.22 ± 4.87) groups (*p* = 0.05). Regarding the SNR, significant intergroup differences were also found for the distal LAD (p = 0.05). The SNR was lowest in the ultra-low PCD-CT group (25.98 ± 9.14), intermediate in the routine PCD-CT group (28.12 ± 10.63), and highest in the routine EID-CT group (29.55 ± 10.56).

**Table 3:** Comparison of objective measurements of CCTA image quality parameters

Variables	Total	Ultra-low in PCD-CT	Routine in PCD-CT	Routine in EID-CT	*p*-value
	(*n* = 292)	(*n* = 96)	(*n* = 99)	(*n* = 97)	
Attenuation (HU)					
AAO	415.98 ± 50.71	414.78 ± 49.89	417.09 ± 52.23	416.04 ± 50.42	0.95
LM	416.44 ± 50.36	421.77 ± 50.79	410.07 ± 48.42	417.66 ± 51.66	0.26
Proximal LAD	417.82 ± 48.78	413.42 ± 49.48	421.87 ± 47.36	418.05 ± 49.65	0.48
Distal LAD	387.92 ± 49.77	373.20 ± 49.58	393.52 ± 49.38	396.72 ± 47.55	0.01
Proximal LCX	418.38 ± 48.54	414.52 ± 46.30	415.18 ± 49.24	425.46 ± 49.69	0.21
Distal LCX	388.35 ± 49.69	374.33 ± 46.83	400.70 ± 50.04	389.84 ± 49.07	< 0.01
Proximal RCA	417.90 ± 50.95	422.40 ± 53.61	417.39 ± 51.80	413.98 ± 47.45	0.52
Distal RCA	387.93 ± 51.11	383.02 ± 54.08	388.42 ± 47.15	392.22 ± 51.97	0.45
Noise					
AAO	17.98 ± 4.36	17.76 ± 4.39	18.01 ± 4.37	18.15 ± 4.37	0.82
LM	17.42 ± 4.14	17.21 ± 3.89	17.42 ± 4.09	17.64 ± 4.44	0.77
Proximal LAD	18.16 ± 4.29	18.24 ± 4.26	17.90 ± 4.47	18.35 ± 4.17	0.75
Distal LAD	15.34 ± 4.40	15.75 ± 4.39	15.40 ± 4.25	14.88 ± 4.55	0.38
Proximal LCX	17.85 ± 4.33	18.00 ± 4.62	18.13 ± 4.32	17.42 ± 4.05	0.48
Distal LCX	15.04 ± 4.51	15.51 ± 4.81	14.31 ± 4.16	15.31 ± 4.50	0.14
Proximal RCA	18.18 ± 4.24	18.17 ± 4.19	18.21 ± 3.89	18.18 ± 4.65	1
Distal RCA	15.34 ± 4.34	15.48 ± 4.13	15.25 ± 4.89	15.28 ± 3.99	0.92
Contrast-to-noise ratio					
AAO	29.37 ± 5.15	29.77 ± 5.18	29.02 ± 5.41	29.33 ± 4.86	0.59
LM	29.54 ± 5.18	29.42 ± 5.45	29.35 ± 5.32	29.86 ± 4.80	0.76
Proximal LAD	29.72 ± 5.20	29.52 ± 5.28	29.29 ± 5.25	30.36 ± 5.04	0.32
Distal LAD	26.51 ± 5.33	25.73 ± 5.43	27.55 ± 5.11	26.24 ± 5.33	0.05
Proximal LCX	29.91 ± 5.22	30.30 ± 5.24	29.73 ± 4.88	29.72 ± 5.56	0.68
Distal LCX	26.80 ± 5.32	27.01 ± 5.40	26.70 ± 5.68	26.69 ± 4.92	0.89
Proximal RCA	29.60 ± 5.16	30.65 ± 5.28	28.96 ± 5.23	29.22 ± 4.87	0.05
Distal RCA	26.46 ± 5.30	27.23 ± 5.46	26.18 ± 5.05	26.00 ± 5.36	0.22
Signal-to-noise ratio					
AAO	29.68 ± 5.34	30.16 ± 5.40	29.48 ± 5.49	29.40 ± 5.13	0.56
LM	29.50 ± 5.40	29.77 ± 5.41	29.45 ± 5.32	29.27 ± 5.49	0.81
Proximal LAD	29.53 ± 5.23	28.97 ± 5.15	29.33 ± 5.22	30.30 ± 5.30	0.19
Distal LAD	27.90 ± 10.21	25.98 ± 9.14	28.12 ± 10.63	29.55 ± 10.56	0.05
Proximal LCX	29.48 ± 5.12	29.81 ± 5.13	29.16 ± 4.97	29.46 ± 5.29	0.68
Distal LCX	28.78 ± 10.99	27.33 ± 11.40	30.80 ± 10.87	28.20 ± 10.53	0.07
Proximal RCA	29.89 ± 5.10	30.12 ± 4.67	29.36 ± 5.23	30.19 ± 5.38	0.45
Distal RCA	27.74 ± 9.75	26.66 ± 8.49	29.03 ± 12.12	27.54 ± 8.10	0.23

*AAO* Ascending aorta, *HU* Hounsfield unit, *LAD* Left descending artery, *LCX* Left circumflex artery, *LM* Left main artery, *RCA* Right coronary artery

### Subjective image quality assessments

Table 4 shows the results of subjective image quality assessment. The distal LAD clarity score was lower in the ultra-low PCD-CT group (2.91 ± 0.81) compared with the routine PCD-CT (3.58 ± 0.50) and routine EID-CT (3.54 ± 0.50) groups (*p* < 0.01). Similarly, distal LCX clarity was reduced in the ultra-low PCD-CT group (3.28 ± 0.66) relative to the routine PCD-CT (3.54 ± 0.50) and routine EID-CT (3.52 ± 0.50) groups (*p* < 0.01). The distal RCA clarity followed the same trend, being lower in the ultra-low PCD-CT group (3.27 ± 0.70) than in the routine PCD-CT (3.57 ± 0.50) and routine EID-CT (3.51 ± 0.50) groups (*p* < 0.01). No significant differences were observed among the groups in coronary arterial contrast, image noise, or beam-hardening artifacts.

**Table 4:** Qualitative assessment of subjective image quality

Variables	Total	Ultra-low in PCD-CT	Routine in PCD-CT	Routine in EID-CT	*p*-value
	(*n* = 292)	(*n* = 96)	(*n* = 99)	(*n* = 97)	
Coronary arterial contrast	3.52 ± 0.50	3.58 ± 0.50	3.46 ± 0.50	3.52 ± 0.50	0.25
Image noise	3.50 ± 0.50	3.48 ± 0.50	3.52 ± 0.50	3.52 ± 0.50	0.85
Beam-hardening artifacts	3.58 ± 0.49	3.61 ± 0.49	3.58 ± 0.50	3.55 ± 0.50	0.62
Distal vessel clarity					
LAD	3.34 ± 0.69	2.91 ± 0.81	3.58 ± 0.50	3.54 ± 0.50	< 0.01
LCX	3.45 ± 0.57	3.28 ± 0.66	3.54 ± 0.50	3.52 ± 0.50	< 0.01
RCA	3.45 ± 0.59	3.27 ± 0.70	3.57 ± 0.50	3.51 ± 0.50	< 0.01

*LAD* Left descending artery, *LCX* Left circumflex artery, *LM* Left main artery, *RCA* Right coronary artery

**Fig. 3:**
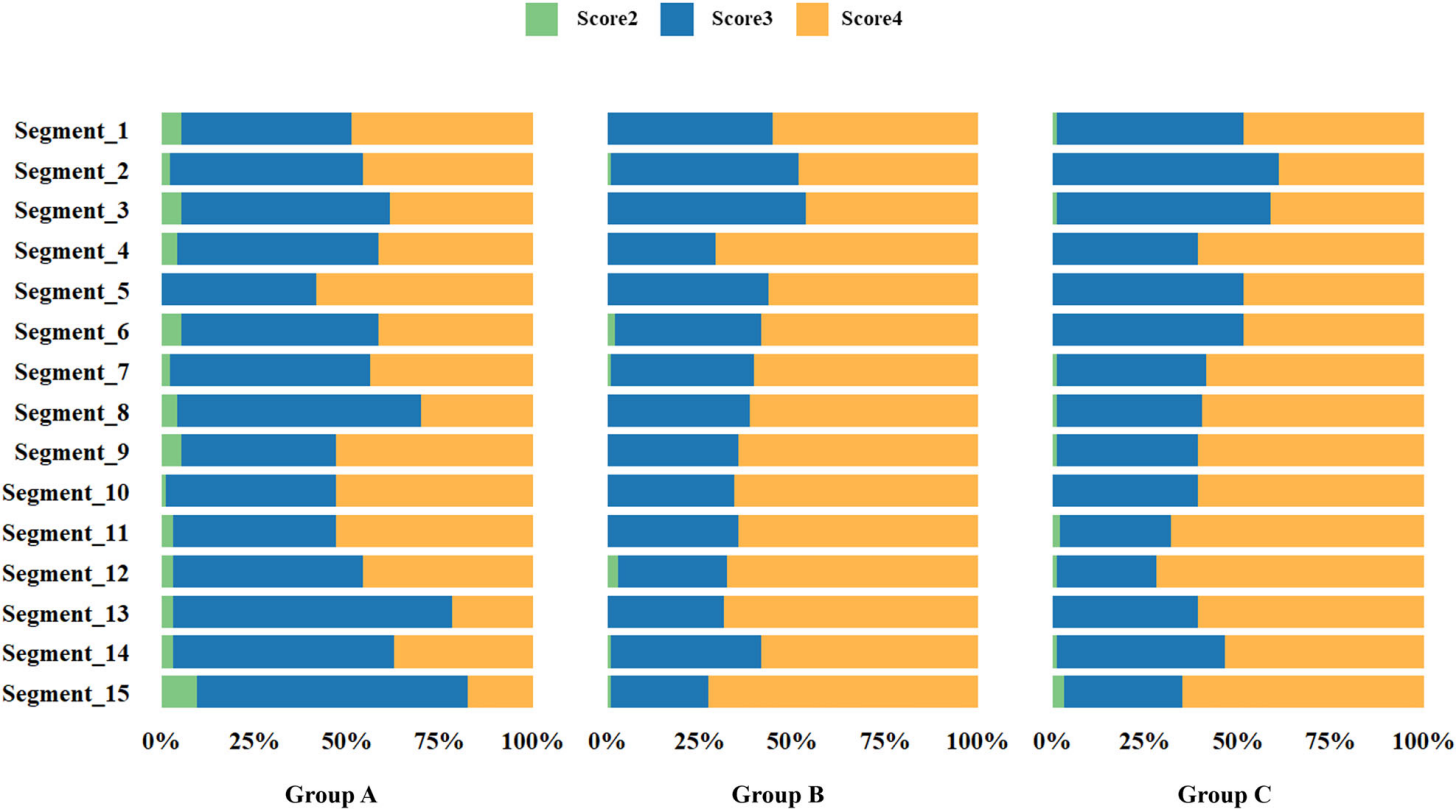
The plot shows the results of subjective image quality on a coronary artery segment basis in different groups. Segment 1, proximal Right Coronary Artery; Segment 2, mid Right Coronary Artery; Segment 3, distal Right Coronary Artery; Segment 4, Posterior descending Artery (Right Coronary Artery); Segment 5, Left Main Coronary Artery; Segment 6, proximal Left Anterior Descending Artery; Segment 7, mid Left Anterior Descending Artery; Segment 8, distal Left Anterior Descending Artery; Segment 9, Diagonal Branches 1; Segment 10, Diagonal Branches 2; Segment 11, proximal Left Circumflex Artery; Segment 12, Obtuse Marginal Branches; Segment 13, distal Left Circumflex Artery; Segment 14, Posterolateral marginal Artery (Left Circumflex Artery); Segment 15, Posterior descending Artery (Left Circumflex Artery)

**Fig. 4:**
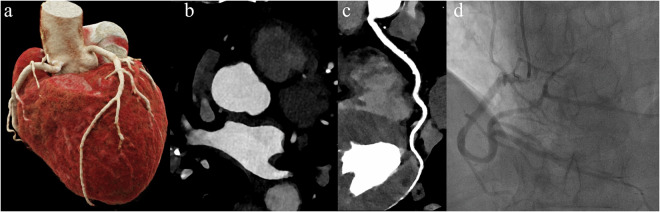
CCTA in a 64-year-old man (HR = 86 bpm, BMI = 26.3 kg/m^2^, Contrast agent flow = 1.8 mL/s, contrast agent volume = 20 mL, CAD-RADS = 1) with chest pain for 3 days. **a** shows the heart cine volume rendering technology images. **b** showed CT value of ascending aorta was 370 HU. **c**, **d** Representative curved multiplanar reformation of the right coronary artery, along with percutaneous coronary angiography, reveals mild stenosis in the mid-segment

**Fig. 5:**
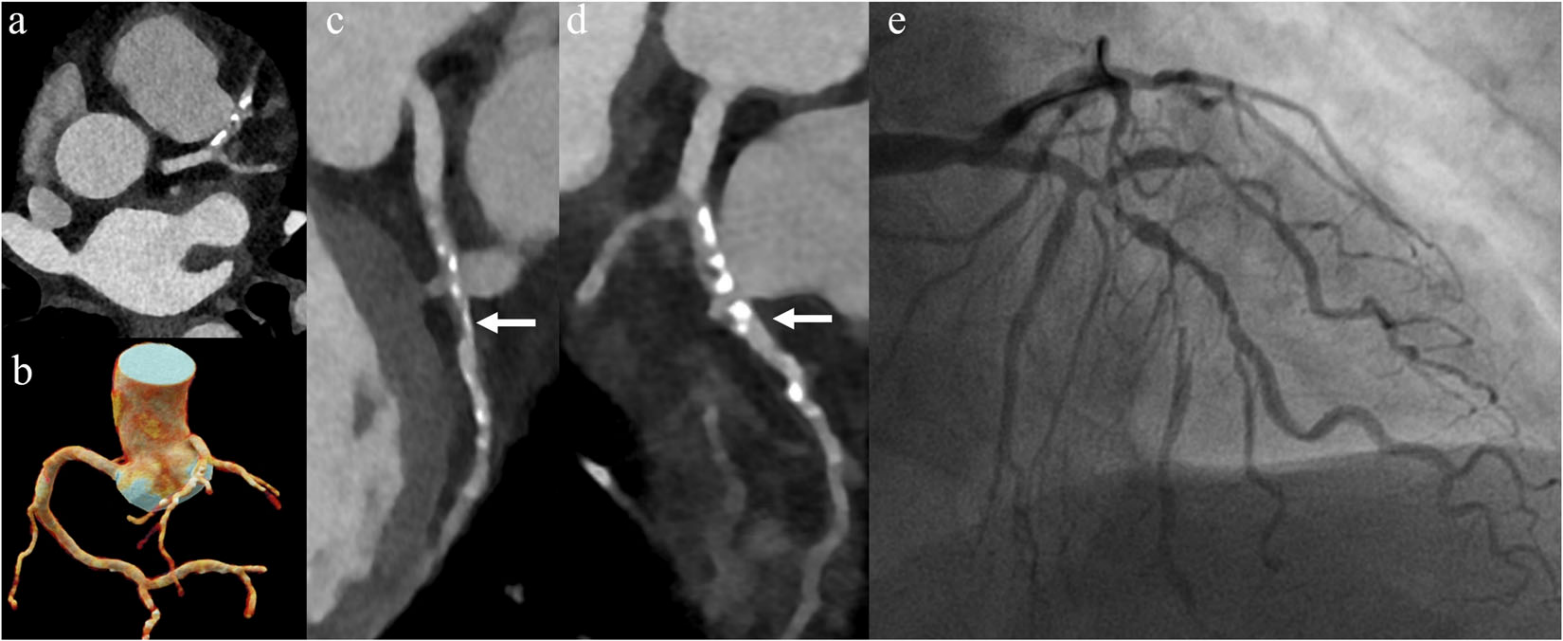
CCTA in a 74-year-old Female (HR = 75 bpm, BMI = 18.3 kg/m^2^, Contrast agent flow = 1.5 mL/s, Contrast agent volume = 15 mL, CAD-RADS = 4) with chest pain for 1 day. **a** shows CT value of AAO was 362 HU. **b** shows the coronary arteries, cine volume rendering technology images. **c**, **d** The anterior descending artery representative curved multiplanar reformation shows the severe stenosis and diffuse calcification in the lumen (White arrow). **e** Percutaneous coronary angiography shows severe stenosis of the left anterior descending artery

Figure 3 illustrates the overall image quality based on the American Heart Association (AHA) coronary segment classification. The ultra-low-dose PCD-CT group demonstrated a higher prevalence of Score 3 and a lower frequency of Score 4, particularly in the distal left circumflex artery (77.1% Score 3) and the posterior descending artery of the left circumflex artery (80.2% Score 3), compared with the routine PCD-CT and EID-CT groups, which predominantly exhibited Score 4 (68.7–72.7%). In the obtuse marginal branch, Score 3 was most frequent in the ultra-low PCD-CT group (54.2%), whereas Score 4 predominated in the routine PCD-CT (67.7%) and EID-CT (72.2%) groups. Figures 4 and  5 present two examples of ultra-low flow rate coronary CTA imaging for two patients.

### Interobserver agreement

Interobserver agreement for subjective assessments of image quality was rated as good to excellent, with ICCs ranging from 0.81 to 0.91. This elevated level of consistency between the two radiologists suggests that the evaluations are both reliable and reproducible. The CT value exhibited an ICC of 0.89, while image noise demonstrated ICCs ranging from 0.87 to 0.96.

## Discussion

This study is the first to evaluate the feasibility of using ultra-low contrast agent flow rates for CCTA with photon-counting detector CT (PCD-CT). Our findings demonstrate that PCD-CT maintains objective image quality comparable to standard contrast injection protocols, even at substantially reduced flow rates. Although subjective image quality scores were slightly lower in the ultra-low contrast group, overall diagnostic performance remained clinically acceptable. These results suggest that ultra-low contrast protocols can be optimized for patients with compromised venous access, providing valuable insights into CT imaging practices.

The direct conversion mechanism of PCD-CT enhances spatial resolution and energy discrimination compared to EID-CT [24], and the resultant reduction in electronic noise with improved SNR is especially beneficial for low-contrast-dose protocols [25, 26]. A key advantage of PCD-CT is its ability to generate VMI, allowing post hoc adjustment of keV levels to enhance vascular contrast through the photoelectric effect at lower energies [27]. These technological advancements enable diagnostic image quality in CCTA even with lower contrast agent doses, as demonstrated in several clinical studies. Oechsner et al achieved high attenuation and CNR in aortoiliac CTA using only 9.8 g of iodine with PCD-CT at 40 keV [28]. Similarly, Rajiah et al reported diagnostic CCTA images acquired in high-pitch multi-energy PCD-CT mode using a contrast agent injected at 5 mL/s [29], and Cundari et al reduced contrast volume by 40% while maintaining diagnostic quality [30]. Based on evidence indicating that 45 keV provides an optimal balance between contrast enhancement and image noise for low-flow CCTA [31–33], we selected this energy level for our ultra-low flow rate PCD-CT protocol.

In our study, we observed that distal coronary segments (especially the distal left anterior descending artery) were less well opacified than under standard injection protocols. This is not surprising: in any CTA, the contrast bolus attenuates as it travels distally, so slower injection (or lower pressure) leads to a steeper drop-off in iodine concentration in small distal branches. In other words, ultra-low flow likely failed to deliver sufficient contrast to fully fill the peripheral coronary tree. This resulted in poorer image quality and occasional “filling defects” in the LAD region. Interestingly, when using a conventional (high-rate) injection protocol, our EID-CT images outperformed PCD-CT in the LAD. One possible explanation is a technical factor: if the heart was not exactly at the isocenter during a high-pitch FLASH acquisition, the PCD detectors might have experienced charge-sharing or “drift” effects in some spectral reconstructions [34, 35]. By contrast, EID-CT acquires a single energy-integrated image and may be less sensitive to slight off-center positioning in fast scans. In practical terms, this means PCD-based keV images can degrade if the source–detector geometry is suboptimal, whereas conventional EID reconstructions remain robust. To counterbalance the inherent iodine signal reduction at ultra-low flow rates, we employed 140 kVp acquisition combined with low-keV VMI reconstruction, leveraging a higher photon flux and superior spectral range to maintain a CNR. Although distal coronary artery segments showed suboptimal opacification in some patients, all subjects in this study achieved image quality sufficient for diagnostic interpretation. These technical adaptations establish a foundation for implementing ultra-low flow protocols in vulnerable patients such as the elderly or those with conditions like chemotherapy-related venous fragility, renal impairment, or dialysis requirements, for whom high-flow contrast injection is contraindicated [36, 37]. Demonstrating that PCD-CT can achieve adequate diagnostic imaging with ultra-low flow rates provides a safer, more accessible CCTA protocol for these patients, potentially reducing risks associated with contrast administration.

By comparing ultra-low contrast flow rates in PCD-CT with standard flow rates in both PCD-CT and EID-CT, we provide evidence that image quality can be maintained while minimizing contrast load. This addresses a critical gap in optimizing CCTA protocols for patients with limited tolerance to high-flow rates. However, our results indicate limitations in patients with significant coronary artery lesions. In the ultra-low flow rate group, distal coronary artery segments were less effectively visualized, particularly in the presence of atherosclerotic plaques or stenoses. Such lesions may impair flow dynamics, hinder contrast propagation and compound the limitations of reduced contrast volume, leading to insufficient opacification in distal vessels.

These findings highlight the importance of tailored protocol selection in PCD-CT CCTA. Patients with poor venous access and minimal coronary artery disease may benefit significantly from the ultra-low contrast protocol, as it provides adequate diagnostic image quality while reducing contrast-related risks. Conversely, patients with significant coronary lesions may require conventional flow rates to ensure sufficient opacification of distal vessels for accurate assessment. To optimize patient outcomes, pre-procedural evaluations should assess coronary disease burden and venous access quality. Interdisciplinary collaboration among cardiologists, radiologists, and technologists can facilitate individualized protocol adjustments, balancing diagnostic efficacy with patient safety.

Despite the promising results, this study has several limitations. First, the relatively small sample size may restrict the generalizability of the findings; larger cohorts are needed to validate these results across diverse patient populations. Second, complete blinding of radiologists was not feasible due to inherent differences between PCD-CT and EID-CT images, as well as the use of virtual monoenergetic imaging, which may have introduced observer bias in subjective assessments. Third, the study focused solely on image quality metrics without evaluating diagnostic performance and radiation dose in detecting coronary artery disease, thereby limiting conclusions about clinical effectiveness. Future research should incorporate diagnostic accuracy metrics, such as sensitivity and specificity, to provide a more comprehensive evaluation of PCD-CT’s clinical utility.

## Conclusion

In conclusion, this study provides robust evidence that ultra-low contrast agent flow rates can be effectively utilized in CCTA imaging with PCD-CT without compromising objective image quality. By addressing a significant research gap and challenging existing controversies, our findings support the innovation and uniqueness of using PCD-CT technology to enhance patient safety and expand the applicability of CCTA. Further research is warranted to validate these results and explore their implications in clinical practice.

## Data Availability

The datasets used and analyzed during the current study are available from the corresponding author on reasonable request.

## Abbreviations

CNR: Contrast-to-noise ratio
EID-CT: Energy-integrating detector CT
LAD: Left anterior descending artery
LCX: Left circumflex artery
LM: Left main artery
PCD-CT: Counting detector CT
RCA: Right coronary artery
SNR: Signal-to-noise ratio
